# Chemical Composition, Apoptotic Activity, and Antiparasitic Effects of *Ferula macrecolea* Essential Oil against *Echinococcus granulosus* Protoscoleces

**DOI:** 10.3390/molecules26040888

**Published:** 2021-02-08

**Authors:** Mohamed S. Alyousif, Hiba Riyadh Al-Abodi, Hamdan Almohammed, Abdullah D. Alanazi, Hossein Mahmoudvand, Marzieh Hakami Shalamzari, Iraj Salimikia

**Affiliations:** 1 Department of Zoology, College of Science, King Saud University, Riyadh 11451, Saudi Arabia; myousifii@ksu.edu.sa; 2 Department of Environment, College of Science, University of Al-Qadisiyah, Al-Diwaniyah 58001, Iraq; hiba.al-abodi@qu.edu.iq; 3 Department of Microbiology and Parasitology, Almaarefa University, Riyadh 11597, Saudi Arabia; hamohammedii@mcst.edu.sa; 4 Department of Biological Science, Faculty of Science and Humanities, Shaqra University, Ad-Dawadimi 11911, Saudi Arabia; aalanazi@su.edu.sa; 5 Alghad International Colleges for Applied Medical Science, Tabuk 47913, Saudi Arabia; 6 Hepatitis Research Center, Lorestan University of Medical Sciences, Khorramabad 6813833946, Iran; dmahmodvand@gmail.com; 7 Student Research Committee, Lorestan University of Medical Sciences, Khorramabad 6813833946, Iran; marziyehakami486@gmail.com; 8 Razi Herbal Medicines Research Center, Lorestan University of Medical Sciences, Khorramabad 6813833946, Iran

**Keywords:** cystic echinococcosis, scolicidal, herbal medicine, apoptosis, caspase activity

## Abstract

Background: Today, the present protoscolicidals used to minimize the serious risks during hydatid cyst surgery are not completely safe and have various adverse side effects. The present study aimed to evaluate the chemical composition and apoptotic activity of *Ferula macrecolea* essential oil (FMEO) as well as its in vitro and ex vivo protoscolicidal effects against hydatid cyst protoscoleces. Methods: Gas chromatography/mass spectrometry (GC/MS) analysis was performed to determine the chemical composition of FMEO. Protoscoleces of hydatid cysts were collected from liver fertile hydatid cysts of infected sheep and were then treated with various concentrations of the essential oil (75, 150, and 300 µL/mL) for 5–60 min in vitro and ex vivo. Then, by using the eosin exclusion test, the viability of the protoscoleces was studied. The caspase-3-like activity of the FMEO-treated protoscoleces was also evaluated through the colorimetric protease assay Sigma Kit based on the manufacturer’s instructions. Results: According to GC/MS, the main constituents of the essential oil were terpinolene (77.72%), n-nonanal (4.47%), and linalool (4.35%), respectively. In vitro, the maximum protoscolicidal activity of FMEO was observed at the concentrations of 150 and 300 µL/mL, such that 100% of the protoscoleces were killed after 30 and 20 min of exposure, respectively. Based on the obtained findings, the results demonstrate that FMEO required a longer time to kill protoscoleces ex vivo; after 12 min of exposure to FMEO, only 13.4% of the protoscoleces remained alive. After 48 h of the treatment of protoscoleces, FMEO, in a dose-dependent manner and at doses of 75, 150, and 300 µL/mL, induced the activation of the caspase enzyme by 24.3, 35.3, and 48.3%, respectively. Conclusions: Our findings demonstrate the potent protoscolicidal effects of FMEO in vitro and ex vivo; however, further studies are required to assess the safety and the efficiency of FMEO as a promising scolicidal agent in a preclinical model and clinical setting.

## 1. Introduction

Cystic echinococcosis (CE) or hydatidosis is well-known as one of the main important parasitic infections caused by the larval stage of the *Echinococcus granulosus* cestode [1]. With respect to the lifecycle of the parasite, humans, as the intermediate host, are generally infected through the ingestion of food, water, vegetables, etc., contaminated with the parasite’s eggs excreted from dogs, the definitive host [2]. While the adult form of *E. granulosus* in the definitive host is not life-threatening, however, the larval stage of the parasite in the intermediate host (humans and some carnivores such as sheep, goats, etc.) can result in severe conditions, even death, by forming hydatid cysts in various vital organs such as the liver, lung, brain, etc. [3]. Although the disease has no clinical signs in the early stages, a wide range of clinical symptoms can appear depending on the location and size of the hydatid cyst [4].

Considering CE treatment, in cases where the cysts are small and inactive, compounds and derivatives of benzimidazole are used as the first-choice treatments, while surgery is the preferred treatment for large and active cysts [5,6]. The rupture of a cyst or leakage of cyst contents (protoscoleces), which leads to secondary infection or the involvement of nearby organs, is the main concern during hydatid cyst surgery [7]. To minimize these risks, various chemical protoscolicidal agents such as hypertonic saline (20%), silver nitrate, and formalin are used [8]. However, recent investigations have reported that these agents are not completely safe and have various adverse side effects including biliary fibrosis, hepatic necrosis, and cirrhosis [9]. For this reason, finding and discovering new scolicidal drugs with high efficiency and minimal toxicity and side effects is a priority for researchers.

From ancient times, medicinal herbs and their derivatives have been broadly used for health promotion and therapy for chronic, as opposed to life-threatening, diseases [10,11,12]. Herbal medicines have also been successfully used in the treatment of a wide range of bacterial, viral, and fungal, as well as parasitic, infections [13,14]. The plants from the genus *Ferula* (Apiaceae family) have nearly 170 species [15]. In traditional medicine, numerous species of the genus *Ferula* have been applied as diuretics, laxatives, antispasmodics, anthelmintics, anticonvulsants, analgesics, etc. [16]. On the other hand, recent studies have demonstrated some pharmacological properties of the plants in this genus including antihypertensive, antinociceptive, anti-inflammatory, antioxidant, antimicrobial, and antifungal effects [17].

There are few studies on the biological and therapeutic activities of *Ferula macrecolea* Boiss.; however, it has been proven that this plant in traditional medicine has various therapeutic properties such as anticonvulsant and antinociceptive effects, being able to remove intestinal parasites, and antihypertensive effects, as well as being able to treat cardiovascular diseases [18]. According to our best knowledge, there is no study on the antiparasitic effects of *F. macrecolea*; therefore, the present study aimed to evaluate the chemical composition, apoptotic activity, and protoscolicidal effects of *F. macrecolea* essential oil (FMEO) and its use against hydatid cyst protoscoleces in vitro and ex vivo.

## 2. Results

### 2.1. GC/MS Analysis

Based on the results obtained in GC/MS, eighteen compounds were identified, making up 98.99% of the total essential oil (Table 1). The major constituents were terpinolene (77.72%), *n*-nonanal (4.47%), and linalool (4.35%), respectively

### 2.2. In Vitro Protoscolicidal Effects of FMEO

**Figure 1:**
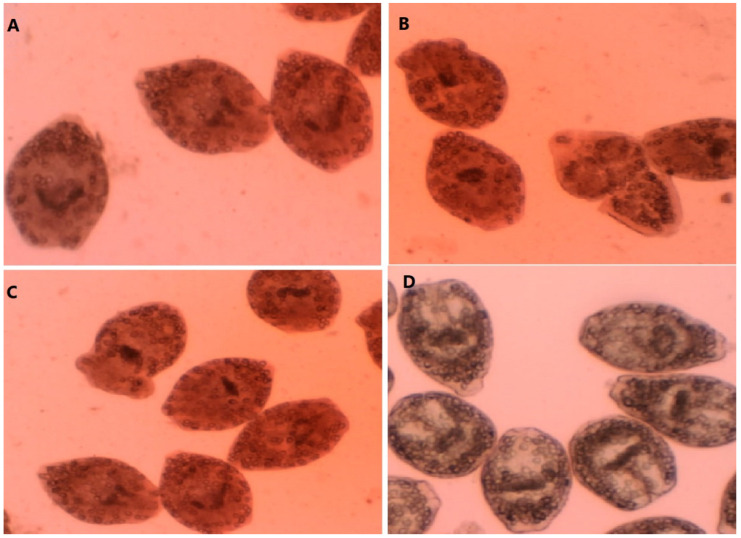
A-C: Dead protoscoleces; *E. granulosus* protoscoleces after exposure to the concentrations of 75 µL/mL (**A**), 150 µL/mL (**B**), and 300 µL/mL (**C**) of *F. macrecolea* essential oil; (**D**) live *E. granulosus* protoscoleces.

As exhibited in Table 2, FMEO at various concentrations had significant protoscolicidal effects in comparison to the control group (*p* < 0.001). The best protoscolicidal activity of FMEO was reported at the concentrations of 150 and 300 µL/mL, where 100% of the protoscoleces were destroyed after 30 and 20 min of treatment, respectively. Meanwhile, the rates of viability in the positive and negative control groups were 0 and 96.7% after 10 and 30 min of treatment, respectively (Figure 1).

### 2.3. Ex Vivo Effect on Protoscoleces

Based on the obtained findings, after adding FMEO at concentrations of 75, 150, and 300 µL/mL into hydatid cysts, the FMEO showed significant protoscolicidal effects. However, the results demonstrate that FMEO needed more time to destroy protoscoleces ex vivo. However, there was no significant difference between the in vitro and ex vivo protoscolicidal effects of FMEO. Table 3 displays the protoscolicidal effects of FMEO ex vivo.

### 2.4. Evaluating the Caspase-3-Like Activity of FMEO-Treated Protoscoleces

**Figure 2:**
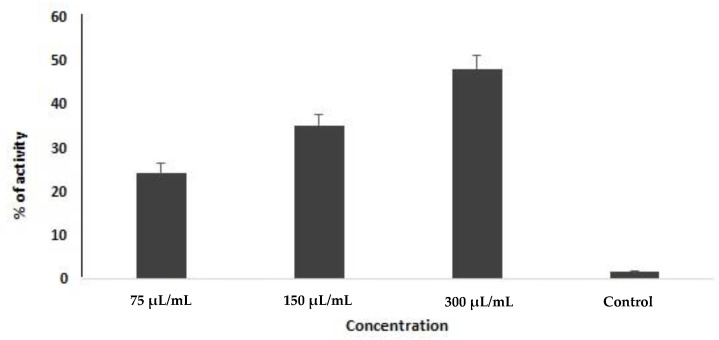
Caspase-3 activity of *E. granulosus* protoscoleces at various concentrations of *F. macrecolea* essential oil. Data shown as mean ± SD from three experiments in duplicate.

In this study, the apoptotic activity of FMEO on *E. granulosus* protoscoleces was determined by the enzymatic activity of caspase-3. To do this, the protoscoleces were exposed to the different concentrations of the FMEO for 2 days, and the percentages of alteration of the activity of the caspase-3 enzyme were evaluated through assessing the concentrations of released NA-*p*. Based on the obtained results, FMEO, in a dose-dependent manner and at doses of 75, 150, and 300 µL/mL, induced the activation of the caspase enzyme by 24.3, 35.3, and 48.3%, respectively (Figure 2).

## 3. Discussion

Over the past centuries, humans have used herbs and their derivatives to treat common infectious diseases, and therefore, some of these herbal medicines are now considered as part of the usual treatment of many infectious diseases [19,20]. Now, various protoscolicidal chemical agents have been recommended for the inactivation of the hydatid cyst protoscoleces during surgery [21,22,23]. Due to the growing resistance and adverse side effects of these chemical agents such as negative effects on liver function, biliary fibrosis, cirrhosis, abdominal pain, diarrhea, nausea, vomiting, etc., the need for a high-performance alternative agent with fewer side effects is strongly felt [9,24]. Although reviews have demonstrated the protoscolicidal activities of the extracts, essential oils, and active compounds of a wide range of medicinal herbs [25,26], according to the World Health Organization (WHO) recommendations, an effective protoscolicidal agent should have some characteristics such as high efficiency in little time, stability in hydatid fluid, low toxicity, high compatibility, low cost, and easy access [27,28].

Based on the previously determined biological properties of *F. macrecolea,* we decided to evaluate the in vitro and ex vivo protoscolicidal effects of *F. macrecolea* essential oil against hydatid cyst protoscoleces. The results show that FMEO had significant protoscolicidal effects compared to the control group (*p* < 0.001). The maximum protoscolicidal activity of FMEO was observed at the concentrations of 150 and 300 µL/mL, such that 100% of the protoscoleces were killed after 30 and 20 min of exposure, respectively. The ex vivo assay showed that after adding FMEO at concentrations of 75, 150, and 300 µL/mL into hydatid cysts, the FMEO showed significant protoscolicidal effects. However, the results demonstrate that FMEO required a longer time to kill protoscoleces ex vivo.

Considering the antimicrobial properties of *Ferula* spp. [29], Asili et al. (2009) demonstrated that the essential oil from the fruits of *F. badrakema* has considerable antibacterial activity against some bacterial and fungal pathogenic strains such as *Staphylococcus aureus*, *Bacillus cereus*, and *Candida albicans* [30]. In a study conducted by Maggi et al. (2009), the antibacterial and antifungal effects of the essential oils from *F. glauca* against *B. subtilis*, *Strptococcus mutans*, *Enterococcus faecalis,* and *Escherichia coli* were demonstrated [31]. Iranshahi et al. (2008) have also reported that the volatile oil of *F. latisecta* had potent antifungal properties acting against some pathogenic dermatophytes including *Trichophyton mentagrophytes*, *T. rubrum*, *T. verrucosom*, *Microsporum canis,* and *M. gypseum* [32]. Ghasemi et al. (2005) demonstrated that the *F. gummosa* fruit’s essential oil had significant antifungal effects against *C. albicans* and *C. kefyr* and antibacterial activity against Gram-positive (*S. aureus, S. epidermis*, and *B. subtilis*) and Gram-negative (*E. coli, Salmonella typhi,* and *Pseudomonas aeruginosa*) bacteria [33]. Rahman et al. (2005) also showed the considerable antifungal effects of *F. assafoetida* seed oil on the stages of the asexual reproduction of some foodborne mold *Aspergillus* species including *A. flavus, A. awamori, A. oryzae, A. niger*, and *A. foetidus* [34].

Considering the antiparasitic effects of *Ferula* species, Iranshahi et al. (2007) demonstrated the antileishmanial effects of a *F. szowitsiana* root acetone extract against *Leishmania major* promastigotes with an IC_50_ value of 11.8 µg/mL [35]. In a study conducted by Esmaeili et al. (2009), the antiplasmodial activity of a *F. oopoda* methanolic extract against the *Plasmodium falciparum* K1 and 3D7 strains was demonstrated, with IC_50_ values of 26.6 and 24.9 µg/mL, respectively [36]. On the other hand, Khanmohammadi et al. (2014) reported that a *F. szowitsiana* methanol extract at the doses of 2 and 3 mg/mL significantly inhibited the growth rate of the *Trichomonas vaginalis* trophozoite after 72 h of incubation, with an LD_50_ value of 0.360 mg/mL [37].

Our findings revealed that according to GC/MS, eighteen compounds were identified, making up 98.99% of the total essential oil. The major constituents were terpinolene (77.72%), n-nonanal (4.47%), and linalool (4.35%), respectively. The study conducted by Rustaiyan et al. (2005) demonstrated that according to GC/MS analysis, the main constituents of *F. macrocolea* essential oil were β-pinene (15.9%), α-pinene (10.4%), and β-caryophyllene (8.6%), respectively [38,39]. Previous studies also showed that the main constituents of *Ferula* spp. essential oil were terpenoid compounds such as α-pinene, β-pinene, terpinolene, α-terpineol, myrcene, etc. [40]. Previous studies have demonstrated that the chemical compositions of the essential oils of plants are rather variable depending on some factors including the collection place, the time of harvest, and the method of extraction [14].

Studies have shown the antibacterial, antifungal, antiviral, and antiparasitic activities of terpenes, terpenoids, and their derivatives against some pathogenic microbial strains [41,42]. Although the precise mechanisms of action of these compounds are not completely understood, some previous investigations have shown that they exhibited antimicrobial effects through the disruption of the cell membrane, inhibition of oxygen consumption, inhibition of virulence factors, etc. [41,42,43].

Today, it has been proven that the induction of apoptosis or programmed cell death is reflected as one of the main possible antimicrobial mechanisms of drugs [44]. Because caspases are the key mechanisms of apoptosis [45], we evaluated the caspase-3 activity in the induced apoptosis in *E. granulous* protoscoleces treated with various concentrations of FMEO. We found that after 48 h of the treatment of the protoscoleces, FMEO, in a dose-dependent manner and at doses of 75, 150, and 300 µL/mL, induced the activation of the caspase enzyme by 24.3, 35.3, and 48.3%, respectively. Thus, the induction of apoptosis can be recommended as one of the likely antimicrobial mechanisms of FMEO.

Considering the cytotoxicity effects, although there is no study on the cytotoxicity of FMEO, the cytotoxic effects of some *Ferula* species such as *F. diversivittata, F. persica, F. ovina, F. badrakema,* and *F. latisecta* and the oleo gum resin of *F. assafoetida* against various cancer and normal cell lines including the HepG2 human liver cancer cell line, the A549 human adenocarcinoma cell line, the HT29 human colorectal adenocarcinoma cell line, the MCF7 breast cancer cell line, the MDBK renal cancer cell line, 4T1 breast cancer cells, and the Vero kidney cell line, with IC_50_ values ranging from 6 to 321 μg/mL, have been evaluated in several studies [45,46,47,48,49,50].

## 4. Materials and Methods

### 4.1. Collecting the Plant Materials

The leaves of *F. macrecolea* were collected from rural regions in August 2020. The plant was identified by a botanist and also, a voucher specimen of the plant material was deposited (RL 1235).

### 4.2. Isolation of the Essential Oil

The air-dried leaves (500 g) were used for hydrodistillation for 3 h by means of an all-glass Clevenger-type device. The obtained essential was dried over anhydrous sodium sulfate and kept in darkness at 4 ˚C in air-tight glass vials closed under nitrogen gas until testing [51,52].

### 4.3. Gas Chromatography/Mass Spectrometry (GC/MS) Analysis of Essential Oil

A Hewlett-Packard 6890 with an HP-5MS column (30 m × 0.25 mm; film thickness, 0.25 mm) was applied to perform the GC analysis. The column temperature was maintained at 50 °C for three minutes, programmed to increase to 290 °C at a rate of 15 °C per min, and kept constant at 290 °C for five minutes. The injector and interface temperatures were 250 and 280 °C, respectively. The flow rate of helium, the carrier gas, was 1 mL/min C.F. The percentages were determined by the electronic integration of the FID peak areas without the use of response factor correction. The linear retention indices for all the components were determined by the coinjection of the samples with a solution containing a homologous series of C8–C24n-alkanes. GC/MS analysis was conducted by means of a Thermoquest-Finnigan gas chromatograph equipped with a fused silica capillary DB-5 column (30 m × 0.25 mm; film thickness, 0.25 mm) coupled with a TRACE mass (Manchester, UK). The range of mass was from 40 to 400 u. The compounds of FMEO were determined through the comparison of their relative retention times and mass spectra with the standard Wiley 2001 library data for the GC/MS system or with data reported in the literature [53].

### 4.4. Collection and Preparation of Protoscoleces

The infected livers of sheep were collected from the slaughterhouse and transferred to the parasitological laboratory. The protoscoleces were collected based on methods described elsewhere [21]. The number of protoscoleces was adjusted to 5 × 10^3^ protoscoleces in a 0.9% NaCl solution with a minimum 90% viability rate.

### 4.5. In Vitro Protoscolicidal Activity

FMEO at the concentrations of 75, 150, and 300 µL/mL was added to 200 µL of the washed protoscoleces (5 × 10^3^ protoscoleces/mL) for 5, 10, 20, and 30 min at 37 °C. After these times, 50 μL of 0.1% eosin stain (Sigma-Aldrich, St. Louis, MO, USA) was added to the protoscoleces, and the mixtures were placed on a glass slide and examined under a light microscope. We determined the viability percentages via calculating the percentage of dead and live protoscoleces among 300 protoscoleces by means of an eosin exclusion experiment [54,55]. This assay is based on the flame cell motility and impermeability to 0.1% eosin solution (1 g of eosin powder in 1000 mL of distilled water); live protoscoleces do not absorb the eosin stain and exhibit characteristic muscular actions and flame cell motion; however, in dead protoscoleces, eosin enters the cell and the protoscoleces become red.

### 4.6. Ex Vivo Protoscolicidal Activity

The livers of sheep that were naturally infected with hydatid cysts were used to evaluate the protoscolicidal activity of FMEO. At first, more than 50% of the hydatid fluid was extracted from the cysts, and then, FMEO was added to the cysts at concentrations of 12.5, 25, 50, and 100 µL/mL. The hydatid fluid was removed from the cyst after 5, 10, 20, and 30 min, stained with 0.1% eosin, and examined under light microscopy for counting [56].

### 4.7. Evaluating the Programmed Cell Death Induced by Caspase-3-Like Activity

Here, the colorimetric protease assay Sigma Kit was applied to assess the apoptotic activity through the evaluation of the caspase-3-like activity of FMEO on protoscoleces. The test was performed based on the measurement of the color spectrophotometric changes created by the release of a molecule (pNA attached to the substrate) by the action of the enzyme caspase-3. After the exposure of protoscoleces to FMEO for 2 days, they were then centrifuged at 600 rpm for 5 min at 4 °C. The sedimented protoscoleces were lysed and then centrifuged at 20,000 rpm for 10 min. In the next step, 5 μg of supernatant was mixed with 85 μL of buffer and 10 μL of caspase-3 (pNA-DEVD-Ac) substrate and was again incubated for 2 h at 37 °C. Finally, the absorption of the samples was read at 405 nm with an ELISA reader.

### 4.8. Statistical Analysis

All the examinations were accomplished in triplicate. The analysis of the data was performed by means of the SPSS 22.0 statistical package (SPSS Inc., Chicago, IL, USA). The one-way ANOVA and descriptive statistics such as frequency calculations were used for data analysis, and the independent-samples *t* test was used for further analysis. *p* < 0.05 was considered statistically significant.

## 5. Conclusions

Our findings demonstrate the potent protoscolicidal effects of FMEO. FMEO has potent scolicidal efficacy in vitro and ex vivo in an intraperitoneal model of the administration of the drug for CE treatment; however, further studies are required to assess the safety and the efficiency of FMEO as a promising scolicidal agent in a preclinical model (in vivo or in an animal model) and clinical setting, not only through intraperitoneal administration but also through different routes such as the oral route. The findings also show that although the possible protoscolicidal mechanisms of FMEO are not obviously understood, the induction of apoptosis through caspases can be considered as one of the main mechanisms.

## Figures and Tables

**Table 1:** Chemical composition of *F. macrecolea* essential oil according to GC/MS analysis.

No.	Composition	RI	Percent (%)
1	α-pinene	936	0.11
2	*p*-cymene	1010	0.23
3	β-phellandrene	1028	1.31
4	Benzeneacetaldehyde	1032	1.35
5	α-thujene	1035	3.92
6	limonene	1036	0.25
7	Methyl carvacrol	1076	1.94
8	Terpinolene	1094	77.72
9	Geijerene	1098	0.58
10	n-nonanal	1102	4.47
11	α-campholenal	1125	0.32
12	Linalool	1139	4.35
13	Camphor	1148	0.24
14	Thuj-3-en-lo-al	1186	0.29
15	Myrtenal	1196	1.22
16	Allo-ocimene	1198	0.42
17	Di-sec-butyl disulfide	1212	0.17
18	Piperiton	1252	0.1
	Total		98.99

**Table 2:** In vitro protoscolicidal effects of different concentrations of *F. macrecolea* essential oil on the *E. granulosus* protoscoleces over 5, 10, 20, and 30 min of incubation. (Mean ± SD).

Concentration (µL/mL)	Time (min)	Mean of Mortality (%)
75	5	36.3 ± 2.51
	10	56.6 ± 3.15
	20	79.6 ± 2.51
	30	98.0 ± 0.0
150	5	58.3 ± 2.51
	10	76.6 ± 3.15
	20	96.6 ± 4.51
	30	100.0 ± 0.0
300	5	71.3 ± 4.15
	10	98.3 ± 4.51
	20	100.0 ± 0.0
	30	100.0 ± 0.0
Normal saline + Tween 20%	5	0.0 ± 0.0
	10	0.0 ± 0.0
	20	1.5 ± 0.5
	30	3.3 ± 0.15
Ag-nitrate	5	71.6 ± 2.88
	10	100.0 ± 0.0
	20	100.0 ± 0.0
	30	100.0 ± 0.0

**Table 3:** Ex vivo protoscolicidal effects of different concentrations of *F. macrecolea* essential oil on the *E. granulosus* protoscoleces over 5, 10, 20, and 30 min of incubation. (Mean ± SD).

Concentration (µL/mL)	Time (min)	Mean of Mortality (%)
300	5	36.6 ± 1.15
	7	58.3 ± 3.51
	12	86.6 ± 4.15
	20	100 ± 0.0
	40	100 ± 0.0
	60	100 ± 0.0
150	5	18.3 ± 1.15
	7	36.3 ± 4.51
	12	53.6 ± 3.15
	20	72.6 ± 4.15
	40	100 ± 0.0
	60	100 ± 0.0
75	5	6.6 ± 0.5
	7	1.15 ± 16.6
	12	32.3 ± 2.88
	20	4.51 ± 54.6
	40	77.3 ± 4.51
	60	93.6 ± 4.51
Normal saline + Tween 20	5	0.0 ± 0.0
	7	1.3 ± 0.57
	12	4.3 ± 0.57
	20	6.6 ± 1.15
	40	7.6 ± 0.57
	60	8.3 ± 1.15
Ag-nitrate	5	42.3 ± 2.88
	7	100 ± 0.0
	12	100 ± 0.0
	20	100 ± 0.0
	40	100 ± 0.0
	60	100 ± 0.0

## Data Availability

All the data generated or analyzed during this study are included in this published article.

## Sample Availability

Samples of the compounds are not available from the authors.

## References

[1] Wen H., Vuitton L., Tuxun T., Li J., Vuitton D.A., Zhang W., McManus D.P. (2019). Echinococcosis: Advances in the 21st Century. Clin. Microbiol. Rev..

[2] Eckert J., Deplazes P. (2004). Biological, Epidemiological, and Clinical Aspects of Echinococcosis, a Zoonosis of Increasing Concern. Clin. Microbiol. Rev..

[3] McManus D.P., Gray D.J., Zhang W., Yang Y. (2012). Diagnosis, treatment, and management of echinococcosis. BMJ.

[4] Geramizadeh B. (2013). Unusual Locations of the Hydatid Cyst: A Review from Iran. Iran. J. Med. Sci..

[5] Gavara C.G.I., López-Andújar R., Ibáñez T.B., Ángel J.M., Herraiz Á.M., Castellanos F.O., Ibars E.P., Rodríguez F.S. (2015). Review of the treatment of liver hydatid cysts. World J. Gastroenterol..

[6] Nazligul Y., Kucukazman M., Akbulut S. (2015). Role of Chemotherapeutic Agents in the Management of Cystic Echinococcosis. Int. Surg..

[7] Tuxun T., Zhang J.-H., Zhao J.-M., Tai Q.-W., Abudurexti M., Ma H.-Z., Wen H. (2014). World review of laparoscopic treatment of liver cystic echinococcosis—914 patients. Int. J. Infect. Dis..

[8] Siracusano A., Teggi A., Ortona E. (2009). Human Cystic Echinococcosis: Old Problems and New Perspectives. Interdiscip. Perspect. Infect. Dis..

[9] Rajabi M.A. (2009). Fatal reactions and methaemoglobinaemia after silver nitrate irrigation of hydatid cyst. Surg. Pract..

[10] Ríos J.L., Recio M.C. (2005). Medicinal plants and antimicrobial activity. J. Ethnopharmacol..

[11] Niazi M., Veiskaramian A., Mokhayeri Y. (2020). Toward nonalcoholic fatty liver treatment; a review on herbal medicine treatment. J. Crit. Rev..

[12] Delfani S., Mohammadrezaei-Khorramabadi R., Ghamari S., Boroujeni R.K., Khodabandeloo N., Khorzoughi M.G., Shahsavari S. (2017). Systematic review for phytotherapy in Streptococcus Mutans. J. Pharm. Sci. Res..

[13] Masoori L., Yazdani S., Rezaei F., Amraei M. (2017). Phytotherapy for Streptococcus viridans. J. Pharm. Sci. Res..

[14] Delfani S., Mohammadrezaei-Khorramabadi R., Abbaszadeh S., Naghdi N., Shahsavari S. (2017). Phytotherapy for Streptococcus pyogenes. J. Pharm. Sci. Res..

[15] Yaqoob U., Nawchoo I.A. (2016). Distribution and taxonomy of Ferula L.: A review. Res. Rev. J. Bot..

[16] Mohammadhosseini M., Venditti A., Sarker S.D., Nahar L., Akbarzadeh A. (2019). The genus Ferula: Ethnobotany, phytochemistry and bioactivities—A review. Ind. Crop. Prod..

[17] Salehi M., Naghavi M.R., Bahmankar M. (2019). A review of Ferula species: Biochemical characteristics, pharmaceutical and industrial applications, and suggestions for biotechnologists. Ind. Crop. Prod..

[18] Zargari A. (1995). Medical Plants.

[19] Moreira D.D., Teixeira S.S., Monteiro M.H., De-Oliveira A.C., Paumgartten F.J. (2014). Traditional use and safety of herbal medicines. Rev. Bras. Farmacogn..

[20] Patra K.C., Pareta S.K., Harwansh R.K., Kumar K.J. (2010). Traditional approaches towards standardization of herbal medicines—A review. J. Pharm. Sci. Technol..

[21] Sharafi S.M., Sefiddashti R.R., Sanei B., Yousefi M., Darani H.Y. (2017). Scolicidal agents for protoscolices of Echinococcus granulosus hydatid cyst: Review of literature. J. Res. Med. Sci..

[22] AlBalawi A.E., Alanazi A.D., Baharvand P., Sepahvand M., Mahmoudvand H. (2020). High Potency of Organic and Inorganic Nanoparticles to Treat Cystic Echinococcosis: An Evidence-Based Review. Nanomaterials.

[23] Mahmoudvand H., Tavakoli Oliaei R., Mirbadie S.R., Kheirandish F., Tavakoli Kareshk A., Ezatpour B., Mahmoudvand H. (2016). Efficacy and safety of Bunium persicum (Boiss) to inactivate protoscoleces during hydatid cyst operations. Surg. Infect..

[24] Junghanss T., Brunetti E., Chiodini P.L., Horton J., Da Silva A.M. (2008). Clinical Management of Cystic Echinococcosis: State of the Art, Problems, and Perspectives. Am. J. Trop. Med. Hyg..

[25] Kohansal M.H., Nourian A., Rahimi M.T., Daryani A., Spotin A., Ahmadpour E. (2017). Natural products applied against hydatid cyst protoscolices: A review of past to present. Acta Trop..

[26] Rostami A., Taheri M., Gholizadeh M., Seyyedtabaei S.J., Raeghi S., Fallahi S. (2016). Scolicidal effect of some herbs on Echinococcus granulosus protoscoleces: A systematic literature review. Herb. Med. J..

[27] WHO Informal Working Group on Echinococcosis (1996). Guidelines for treatment of cystic and alveolar echinococcosis in humans. Bull. World Health Organ..

[28] Eckert J., Gemmell M.A., Meslin F.X., Pawlowski Z.S., World Health Organization (2001). WHO/OIE Manual on Echinococcosis in Humans and Animals: A Public Health Problem of Global Concern.

[29] Boghrati Z., Iranshahi M. (2019). Ferula species: A rich source of antimicrobial compounds. J. Herb. Med..

[30] Asili J., Sahebkar A., Bazzaz B.S.F., Sharifi S., Iranshahi M. (2009). Identification of Essential Oil Components ofFerula badrakemaFruits by GC-MS and13C-NMR Methods and Evaluation of its Antimicrobial Activity. J. Essent. Oil Bear. Plants.

[31] Maggi F., Cecchini C., Cresci A., Coman M., Tirillini B., Sagratini G., Papa F. (2009). Chemical composition and antimicrobial activity of the essential oil from Ferula glauca L. (F. communis L. subsp. glauca) growing in Marche (central Italy). Fitoterapia.

[32] Iranshahi M., Fata A., Emami B., Shahri B.M.J., Bazzaz B.S.F. (2008). In Vitro Antifungal Activity of Polysulfides-Rich Essential Oil of Ferula Latisecta Fruits against Human Pathogenic Dermatophytes. Nat. Prod. Commun..

[33] Ghasemi Y., Faridi P., Mehregan I., Mohagheghzadeh A. (2005). Ferula gummosa Fruits: An Aromatic Antimicrobial Agent. Chem. Nat. Compd..

[34] Rahman I.R., Gul S.H., Odhano E.A. (2008). Antimicrobial activities of Ferula assafoetida oil against gram positive and gram negative bacteria. Am. Eurasian J. Agric..

[35] Iranshahi M., Arfa P., Ramezani M., Jaafari M.R., Sadeghian H., Bassarello C., Piacente S., Pizza C. (2007). Sesquiterpene coumarins from Ferula szowitsiana and in vitro antileishmanial activity of 7-prenyloxycoumarins against promastigotes. Phytochemistry.

[36] Esmaeili S., Naghibi F., Mosaddegh M., Sahranavard S., Ghafari S., Abdullah N.R. (2009). Screening of antiplasmodial properties among some traditionally used Iranian plants. J. Ethnopharmacol..

[37] Khanmohammadi M., Ganji S., Rad S.R. (2014). Anti-protozoan Effects of Methanol Extracts of the Ferula szowitsiana on the Trichomonas Vaginalis Trophozoites in vitro. Int. J. Women Health Rep. Sci..

[38] Dhifi W., Bellili S., Jazi S., Bahloul N., Mnif W. (2016). Essential Oils’ Chemical Characterization and Investigation of Some Biological Activities: A Critical Review. Medcines.

[39] Rustaiyan A., Nadimi M., Mazloomifar H., Massudi S. (2005). Composition of the Essential Oil ofFerula macrocolea(Boiss.) Boiss. from Iran. J. Essent. Oil Res..

[40] Sahebkar A., Iranshahi M. (2011). Volatile Constituents of the Genus Ferula (Apiaceae): A Review. J. Essent. Oil Bear. Plants.

[41] Guimarães A.C., Meireles L.M., Lemos M.F., Guimarães M.C.C., Endringer D.C., Fronza M., Scherer R. (2019). Antibacterial Activity of Terpenes and Terpenoids Present in Essential Oils. Molecules.

[42] Mahizan N.A., Yang S.-K., Moo C.-L., Song A.A.-L., Chong C.-M., Chong C.W., Abushelaibi A., Lim S.H.E., Song L.K. (2019). Terpene Derivatives as a Potential Agent against Antimicrobial Resistance (AMR) Pathogens. Molecules.

[43] Srivastava A.K., Singh V.K. (2019). Biological action of essential oils (terpenes). Int. J. Biol. Med. Res..

[44] Elmore S. (2007). Apoptosis: A Review of Programmed Cell Death. Toxicol. Pathol..

[45] Kumar S. (2006). Caspase function in programmed cell death. Cell Death Differ..

[46] Hosseinzadeh N., Shomali T., Hosseinzadeh S., Fard F.R., Jalaei J., Fazeli M. (2020). Cytotoxic activity of Ferula persica gum essential oil on murine colon carcinoma (CT26) and Vero cell lines. J. Essent. Oil Res..

[47] Iranshahi M., Rezaee R., Najafi M.N., Haghbin A., Kasaian J. (2018). Cytotoxic activity of the genus Ferula (Apiaceae) and its bioactive constituents. Avicenna J. Phytomed..

[48] Valiahdi S.M., Iranshahi M., Sahebkar A. (2013). Cytotoxic activities of phytochemicals from Ferula species. DARU J. Pharm. Sci..

[49] Bagheri S.M., Asl A.A., Shams A., Mirghanizadeh-Bafghi S.A., Hafizibarjin Z. (2017). Evaluation of Cytotoxicity Effects of Oleo-Gum-Resin and Its Essential Oil of Ferula assa-foetida and Ferulic Acid on 4T1 Breast Cancer Cells. Indian J. Med. Paediatr. Oncol..

[50] Esmaeili S., Hajimehdipoor H., Ramezani A., Mosaddegh M. (2012). The Cytotoxic Effects of Ferula Persica var. Persica and Ferula Hezarlalehzarica against HepG2, A549, HT29, MCF7 and MDBK Cell Lines. Iran. J. Pharm. Sci..

[51] Mahmoudvand H., Nadri S., Jahanbakhsh S. (2016). Nectaroscordum tripedale essential oil: Protoscolicidal effects against hydatid cyst protoscoleces. Der Pharma Chem..

[52] Ashrafi B., Ramak P., Ezatpour B., Talei G.R. (2019). Biological Activity and Chemical Composition of the Essential Oil of *Nepeta cataria* L.. J. Res. Pharm..

[53] Adams R.P. (2004). Identification of Essential Oil Components by Gas Chromatography/Mass Spectroscopy.

[54] Moazeni M., Larki S., Pirmoradi G., Rahdar M. (2014). Scolicidal effect of the aromatic water of Zataria multiflora: An in vitro study. Comp. Clin. Pathol..

[55] Moazeni M., Hosseini S., Al-Qanbar M., Alavi A., Khazraei H. (2019). In vitro evaluation of the protoscolicidal effect of Eucalyptus globulus essential oil on protoscolices of hydatid cyst compared with hypertonic saline, povidone iodine and silver nitrate. J. Visc. Surg..

[56] Niazi M., Saki M., Sepahvand M., Jahanbakhsh S., Khatami M., Beyranvand M. (2019). In vitro and ex vivo scolicidal effects of Olea europaea L. to inactivate the protoscolecs during hydatid cyst surgery. Ann. Med. Surg..

